# Supplementary material for: Resolving the full spectrum of human genome variation using Linked-Reads
Source: Genome Res. 2019 Apr;29(4):635–45. doi: 10.1101/gr.234443.118 (PMC6442396; doi:10.1101/gr.234443.118)
Supplement: Supplemental Material [file supp_gr.234443.118_Supplemental_longranger-2.2.zip › longranger-2.2.tar.bz2!/lr2.2-public/lib/stan/cmdstan/stan/doc/api/html/command_8hpp_source.html]

Stan: src/stan/common/command.hpp Source File


|  |  |
| --- | --- |
|  | Stan  2.5.0  probability, sampling & optimization |


- Main Page
- Related Pages
- Namespaces
- Classes
- Files

- File List
- File Members

All Classes Namespaces Files Functions Variables Typedefs Enumerations Enumerator Friends Macros Pages

- src
- stan
- common

command.hpp

Go to the documentation of this file.

1 #ifndef STAN\_\_COMMON\_\_COMMAND\_HPP

2 #define STAN\_\_COMMON\_\_COMMAND\_HPP

3

4 #include <fstream>

5 #include <stdexcept>

6

7 #include <boost/date\_time/posix\_time/posix\_time\_types.hpp>

8 #include <boost/math/special\_functions/fpclassify.hpp>

9 #include <boost/random/additive\_combine.hpp> // L'Ecuyer RNG

10 #include <boost/random/uniform\_real\_distribution.hpp>

11

12 #include <stan/version.hpp>

13 #include <stan/io/cmd\_line.hpp>

14 #include <stan/io/dump.hpp>

15 #include <stan/io/json.hpp>

16 #include <stan/io/mcmc\_writer.hpp>

17

18 #include <stan/gm/arguments/argument\_parser.hpp>

19 #include <stan/gm/arguments/arg\_id.hpp>

20 #include <stan/gm/arguments/arg\_data.hpp>

21 #include <stan/gm/arguments/arg\_init.hpp>

22 #include <stan/gm/arguments/arg\_random.hpp>

23 #include <stan/gm/arguments/arg\_output.hpp>

24

25 #include <stan/mcmc/fixed\_param\_sampler.hpp>

26 #include <stan/mcmc/hmc/static/adapt\_unit\_e\_static\_hmc.hpp>

27 #include <stan/mcmc/hmc/static/adapt\_diag\_e\_static\_hmc.hpp>

28 #include <stan/mcmc/hmc/static/adapt\_dense\_e\_static\_hmc.hpp>

29 #include <stan/mcmc/hmc/nuts/adapt\_unit\_e\_nuts.hpp>

30 #include <stan/mcmc/hmc/nuts/adapt\_diag\_e\_nuts.hpp>

31 #include <stan/mcmc/hmc/nuts/adapt\_dense\_e\_nuts.hpp>

32

33 #include <stan/model/util.hpp>

34

35 #include <stan/optimization/newton.hpp>

36 #include <stan/optimization/bfgs.hpp>

37

38 #include <stan/common/write\_iteration\_csv.hpp>

39 #include <stan/common/write\_iteration.hpp>

40 #include <stan/common/write\_stan.hpp>

41 #include <stan/common/write\_model.hpp>

42 #include <stan/common/write\_error\_msg.hpp>

43 #include <stan/common/do\_print.hpp>

44 #include <stan/common/do\_bfgs\_optimize.hpp>

45 #include <stan/common/print\_progress.hpp>

46 #include <stan/common/run\_markov\_chain.hpp>

47 #include <stan/common/warmup.hpp>

48 #include <stan/common/sample.hpp>

49 #include <stan/common/init\_static\_hmc.hpp>

50 #include <stan/common/init\_nuts.hpp>

51 #include <stan/common/init\_adapt.hpp>

52 #include <stan/common/init\_windowed\_adapt.hpp>

53 #include <stan/common/recorder/csv.hpp>

54 #include <stan/common/recorder/messages.hpp>

55 #include <stan/common/initialize\_state.hpp>

56 #include <stan/common/context\_factory.hpp>

57

58 namespace stan {

59

60  namespace common {

61

62  struct NoOpFunctor {

63  void operator()() { }

64  };

65

66  template <class Model>

67  int command(int argc, const char\* argv[]) {

68

69  std::vector<stan::gm::argument\*> valid\_arguments;

70  valid\_arguments.push\_back(new stan::gm::arg\_id());

71  valid\_arguments.push\_back(new stan::gm::arg\_data());

72  valid\_arguments.push\_back(new stan::gm::arg\_init());

73  valid\_arguments.push\_back(new stan::gm::arg\_random());

74  valid\_arguments.push\_back(new stan::gm::arg\_output());

75

76  stan::gm::argument\_parser parser(valid\_arguments);

77  int err\_code = parser.parse\_args(argc, argv, &std::cout, &std::cout);

78

79  if (err\_code != 0) {

80  std::cout << "Failed to parse arguments, terminating Stan" << std::endl;

81  return err\_code;

82  }

83

84  if (parser.help\_printed())

85  return err\_code;

86

87  // Identification

88  unsigned int id = dynamic\_cast<stan::gm::int\_argument\*>(parser.arg("id"))->value();

89

91  // Random number generator //

93

94  unsigned int random\_seed = 0;

95  stan::gm::u\_int\_argument\* random\_arg

96  = dynamic\_cast<stan::gm::u\_int\_argument\*>(parser.arg("random")->arg("seed"));

97

98  if (random\_arg->is\_default()) {

99  random\_seed = (boost::posix\_time::microsec\_clock::universal\_time() -

100  boost::posix\_time::ptime(boost::posix\_time::min\_date\_time))

101  .total\_milliseconds();

102

103  random\_arg->set\_value(random\_seed);

104

105  } else {

106  random\_seed = random\_arg->value();

107  }

108

109  typedef boost::ecuyer1988 rng\_t; // (2\*\*50 = 1T samples, 1000 chains)

110  rng\_t base\_rng(random\_seed);

111

112  // Advance generator to avoid process conflicts

113  static boost::uintmax\_t DISCARD\_STRIDE = static\_cast<boost::uintmax\_t>(1) << 50;

114  base\_rng.discard(DISCARD\_STRIDE \* (id - 1));

115

117  // Input/Output //

119

120  // Data input

121  std::string data\_file

122  = dynamic\_cast<stan::gm::string\_argument\*>(parser.arg("data")->arg("file"))->value();

123

124  std::fstream data\_stream(data\_file.c\_str(),

125  std::fstream::in);

126  stan::io::dump data\_var\_context(data\_stream);

127  data\_stream.close();

128

129  // Sample output

130  std::string output\_file = dynamic\_cast<stan::gm::string\_argument\*>(

131  parser.arg("output")->arg("file"))->value();

132  std::fstream\* output\_stream = 0;

133  if (output\_file != "") {

134  output\_stream = new std::fstream(output\_file.c\_str(),

135  std::fstream::out);

136  }

137

138  // Diagnostic output

139  std::string diagnostic\_file = dynamic\_cast<stan::gm::string\_argument\*>(

140  parser.arg("output")->arg("diagnostic\_file"))->value();

141

142  std::fstream\* diagnostic\_stream = 0;

143  if (diagnostic\_file != "") {

144  diagnostic\_stream = new std::fstream(diagnostic\_file.c\_str(),

145  std::fstream::out);

146  }

147

148  // Refresh rate

149  int refresh = dynamic\_cast<stan::gm::int\_argument\*>(

150  parser.arg("output")->arg("refresh"))->value();

151

153  // Initialize Model //

155

156  Model model(data\_var\_context, &std::cout);

157

158  Eigen::VectorXd cont\_params = Eigen::VectorXd::Zero(model.num\_params\_r());

159

160  parser.print(&std::cout);

161  std::cout << std::endl;

162

163  if (output\_stream) {

164  write\_stan(output\_stream, "#");

165  write\_model(output\_stream, model.model\_name(), "#");

166  parser.print(output\_stream, "#");

167  }

168

169  if (diagnostic\_stream) {

170  write\_stan(diagnostic\_stream, "#");

171  write\_model(diagnostic\_stream, model.model\_name(), "#");

172  parser.print(diagnostic\_stream, "#");

173  }

174

175  std::string init = dynamic\_cast<stan::gm::string\_argument\*>(

176  parser.arg("init"))->value();

177

178  dump\_factory var\_context\_factory;

179  if (!initialize\_state<dump\_factory>

180  (init, cont\_params, model, base\_rng, &std::cout,

181  var\_context\_factory))

182  return stan::gm::error\_codes::SOFTWARE;

183

185  // Model Diagnostics //

187

188  if (parser.arg("method")->arg("diagnose")) {

189

190  std::vector<double> cont\_vector(cont\_params.size());

191  for (int i = 0; i < cont\_params.size(); ++i)

192  cont\_vector.at(i) = cont\_params(i);

193  std::vector<int> disc\_vector;

194

195  stan::gm::list\_argument\* test = dynamic\_cast<stan::gm::list\_argument\*>

196  (parser.arg("method")->arg("diagnose")->arg("test"));

197

198  if (test->value() == "gradient") {

199  std::cout << std::endl << "TEST GRADIENT MODE" << std::endl;

200

201  double epsilon = dynamic\_cast<stan::gm::real\_argument\*>

202  (test->arg("gradient")->arg("epsilon"))->value();

203

204  double error = dynamic\_cast<stan::gm::real\_argument\*>

205  (test->arg("gradient")->arg("error"))->value();

206

207  int num\_failed

208  = stan::model::test\_gradients<true,true>(model,cont\_vector, disc\_vector,

209  epsilon, error, std::cout);

210

211  if (output\_stream) {

212  num\_failed

213  = stan::model::test\_gradients<true,true>(model,cont\_vector, disc\_vector,

214  epsilon, error, \*output\_stream);

215  }

216

217  if (diagnostic\_stream) {

218  num\_failed

219  = stan::model::test\_gradients<true,true>(model,cont\_vector, disc\_vector,

220  epsilon, error, \*diagnostic\_stream);

221  }

222

223  (void) num\_failed; // FIXME: do something with the number failed

224

225  return stan::gm::error\_codes::OK;

226  }

227

228  }

229

231  // Optimization Algorithms //

233

234  if (parser.arg("method")->arg("optimize")) {

235  std::vector<double> cont\_vector(cont\_params.size());

236  for (int i = 0; i < cont\_params.size(); ++i)

237  cont\_vector.at(i) = cont\_params(i);

238  std::vector<int> disc\_vector;

239

240  stan::gm::list\_argument\* algo = dynamic\_cast<stan::gm::list\_argument\*>

241  (parser.arg("method")->arg("optimize")->arg("algorithm"));

242

243  int num\_iterations = dynamic\_cast<stan::gm::int\_argument\*>(

244  parser.arg("method")->arg("optimize")->arg("iter"))->value();

245

246  bool save\_iterations

247  = dynamic\_cast<stan::gm::bool\_argument\*>(parser.arg("method")

248  ->arg("optimize")

249  ->arg("save\_iterations"))->value();

250  if (output\_stream) {

251  std::vector<std::string> names;

252  names.push\_back("lp\_\_");

253  model.constrained\_param\_names(names,true,true);

254

255  (\*output\_stream) << names.at(0);

256  for (size\_t i = 1; i < names.size(); ++i) {

257  (\*output\_stream) << "," << names.at(i);

258  }

259  (\*output\_stream) << std::endl;

260  }

261

262  double lp(0);

263  int return\_code = stan::gm::error\_codes::CONFIG;

264  if (algo->value() == "newton") {

265  std::vector<double> gradient;

266  try {

267  lp = model.template log\_prob<false, false>(cont\_vector, disc\_vector, &std::cout);

268  } catch (const std::exception& e) {

269  write\_error\_msg(&std::cout, e);

270  lp = -std::numeric\_limits<double>::infinity();

271  }

272

273  std::cout << "initial log joint probability = " << lp << std::endl;

274  if (output\_stream && save\_iterations) {

275  write\_iteration(\*output\_stream, model, base\_rng,

276  lp, cont\_vector, disc\_vector);

277  }

278

279  double lastlp = lp \* 1.1;

280  int m = 0;

281  std::cout << "(lp - lastlp) / lp > 1e-8: " << ((lp - lastlp) / fabs(lp)) << std::endl;

282  while ((lp - lastlp) / fabs(lp) > 1e-8) {

283

284  lastlp = lp;

285  lp = stan::optimization::newton\_step(model, cont\_vector, disc\_vector);

286  std::cout << "Iteration ";

287  std::cout << std::setw(2) << (m + 1) << ". ";

288  std::cout << "Log joint probability = " << std::setw(10) << lp;

289  std::cout << ". Improved by " << (lp - lastlp) << ".";

290  std::cout << std::endl;

291  std::cout.flush();

292  m++;

293

294  if (output\_stream && save\_iterations) {

295  write\_iteration(\*output\_stream, model, base\_rng,

296  lp, cont\_vector, disc\_vector);

297  }

298

299  }

300  return\_code = stan::gm::error\_codes::OK;

301  } else if (algo->value() == "bfgs") {

302  NoOpFunctor callback;

303  typedef stan::optimization::BFGSLineSearch<Model,stan::optimization::BFGSUpdate\_HInv<> > Optimizer;

304  Optimizer bfgs(model, cont\_vector, disc\_vector, &std::cout);

305

306  bfgs.\_ls\_opts.alpha0 = dynamic\_cast<stan::gm::real\_argument\*>(

307  algo->arg("bfgs")->arg("init\_alpha"))->value();

308  bfgs.\_conv\_opts.tolAbsF = dynamic\_cast<stan::gm::real\_argument\*>(

309  algo->arg("bfgs")->arg("tol\_obj"))->value();

310  bfgs.\_conv\_opts.tolRelF = dynamic\_cast<stan::gm::real\_argument\*>(

311  algo->arg("bfgs")->arg("tol\_rel\_obj"))->value();

312  bfgs.\_conv\_opts.tolAbsGrad = dynamic\_cast<stan::gm::real\_argument\*>(

313  algo->arg("bfgs")->arg("tol\_grad"))->value();

314  bfgs.\_conv\_opts.tolRelGrad = dynamic\_cast<stan::gm::real\_argument\*>(

315  algo->arg("bfgs")->arg("tol\_rel\_grad"))->value();

316  bfgs.\_conv\_opts.tolAbsX = dynamic\_cast<stan::gm::real\_argument\*>(

317  algo->arg("bfgs")->arg("tol\_param"))->value();

318  bfgs.\_conv\_opts.maxIts = num\_iterations;

319

320  return\_code = do\_bfgs\_optimize(model,bfgs, base\_rng,

321  lp, cont\_vector, disc\_vector,

322  output\_stream, &std::cout,

323  save\_iterations, refresh,

324  callback);

325  } else if (algo->value() == "lbfgs") {

326  NoOpFunctor callback;

327  typedef stan::optimization::BFGSLineSearch<Model,stan::optimization::LBFGSUpdate<> > Optimizer;

328  Optimizer bfgs(model, cont\_vector, disc\_vector, &std::cout);

329

330  bfgs.get\_qnupdate().set\_history\_size(dynamic\_cast<gm::int\_argument\*>(

331  algo->arg("lbfgs")->arg("history\_size"))->value());

332  bfgs.\_ls\_opts.alpha0 = dynamic\_cast<gm::real\_argument\*>(

333  algo->arg("lbfgs")->arg("init\_alpha"))->value();

334  bfgs.\_conv\_opts.tolAbsF = dynamic\_cast<gm::real\_argument\*>(

335  algo->arg("lbfgs")->arg("tol\_obj"))->value();

336  bfgs.\_conv\_opts.tolRelF = dynamic\_cast<gm::real\_argument\*>(

337  algo->arg("lbfgs")->arg("tol\_rel\_obj"))->value();

338  bfgs.\_conv\_opts.tolAbsGrad = dynamic\_cast<gm::real\_argument\*>(

339  algo->arg("lbfgs")->arg("tol\_grad"))->value();

340  bfgs.\_conv\_opts.tolRelGrad = dynamic\_cast<gm::real\_argument\*>(

341  algo->arg("lbfgs")->arg("tol\_rel\_grad"))->value();

342  bfgs.\_conv\_opts.tolAbsX = dynamic\_cast<gm::real\_argument\*>(

343  algo->arg("lbfgs")->arg("tol\_param"))->value();

344  bfgs.\_conv\_opts.maxIts = num\_iterations;

345

346  return\_code = do\_bfgs\_optimize(model,bfgs, base\_rng,

347  lp, cont\_vector, disc\_vector,

348  output\_stream, &std::cout,

349  save\_iterations, refresh,

350  callback);

351  } else {

352  return\_code = stan::gm::error\_codes::CONFIG;

353  }

354

355  if (output\_stream) {

356  write\_iteration(\*output\_stream, model, base\_rng,

357  lp, cont\_vector, disc\_vector);

358  output\_stream->close();

359  delete output\_stream;

360  }

361  return return\_code;

362  }

363

365  // Sampling Algorithms //

367

368  if (parser.arg("method")->arg("sample")) {

369

370  // Check timing

371  clock\_t start\_check = clock();

372

373  double init\_log\_prob;

374  Eigen::VectorXd init\_grad = Eigen::VectorXd::Zero(model.num\_params\_r());

375

376  stan::model::gradient(model, cont\_params, init\_log\_prob, init\_grad, &std::cout);

377

378  clock\_t end\_check = clock();

379  double deltaT = (double)(end\_check - start\_check) / CLOCKS\_PER\_SEC;

380

381  std::cout << std::endl;

382  std::cout << "Gradient evaluation took " << deltaT << " seconds" << std::endl;

383  std::cout << "1000 transitions using 10 leapfrog steps per transition would take "

384  << 1e4 \* deltaT << " seconds." << std::endl;

385  std::cout << "Adjust your expectations accordingly!" << std::endl << std::endl;

386  std::cout << std::endl;

387

388  stan::common::recorder::csv sample\_recorder(output\_stream, "# ");

389  stan::common::recorder::csv diagnostic\_recorder(diagnostic\_stream, "# ");

390  stan::common::recorder::messages message\_recorder(&std::cout, "# ");

391

392  stan::io::mcmc\_writer<Model,

393  stan::common::recorder::csv, stan::common::recorder::csv,

394  stan::common::recorder::messages>

395  writer(sample\_recorder, diagnostic\_recorder, message\_recorder, &std::cout);

396

397  // Sampling parameters

398  int num\_warmup = dynamic\_cast<stan::gm::int\_argument\*>(

399  parser.arg("method")->arg("sample")->arg("num\_warmup"))->value();

400

401  int num\_samples = dynamic\_cast<stan::gm::int\_argument\*>(

402  parser.arg("method")->arg("sample")->arg("num\_samples"))->value();

403

404  int num\_thin = dynamic\_cast<stan::gm::int\_argument\*>(

405  parser.arg("method")->arg("sample")->arg("thin"))->value();

406

407  bool save\_warmup = dynamic\_cast<stan::gm::bool\_argument\*>(

408  parser.arg("method")->arg("sample")->arg("save\_warmup"))->value();

409

410  stan::mcmc::sample s(cont\_params, 0, 0);

411

412  double warmDeltaT;

413  double sampleDeltaT;

414

415  // Sampler

416  stan::mcmc::base\_mcmc\* sampler\_ptr = 0;

417

418  stan::gm::list\_argument\* algo = dynamic\_cast<stan::gm::list\_argument\*>

419  (parser.arg("method")->arg("sample")->arg("algorithm"));

420

421  stan::gm::categorical\_argument\* adapt = dynamic\_cast<stan::gm::categorical\_argument\*>(

422  parser.arg("method")->arg("sample")->arg("adapt"));

423  bool adapt\_engaged = dynamic\_cast<stan::gm::bool\_argument\*>(adapt->arg("engaged"))->value();

424

425  if (model.num\_params\_r() == 0 && algo->value() != "fixed\_param") {

426  std::cout

427  << "Must use algorithm=fixed\_param for model that has no parameters."

428  << std::endl;

429  return -1;

430  }

431

432  if (algo->value() == "fixed\_param") {

433

434  sampler\_ptr = new stan::mcmc::fixed\_param\_sampler();

435

436  adapt\_engaged = false;

437

438  if (num\_warmup != 0) {

439  std::cout << "Warning: warmup will be skipped for the fixed parameter sampler!" << std::endl;

440  num\_warmup = 0;

441  }

442

443  } else if (algo->value() == "rwm") {

444

445  std::cout << algo->arg("rwm")->description() << std::endl;

446  return 0;

447

448  } else if (algo->value() == "hmc") {

449

450  int engine\_index = 0;

451  stan::gm::list\_argument\* engine

452  = dynamic\_cast<stan::gm::list\_argument\*>(algo->arg("hmc")->arg("engine"));

453  if (engine->value() == "static") {

454  engine\_index = 0;

455  } else if (engine->value() == "nuts") {

456  engine\_index = 1;

457  }

458

459  int metric\_index = 0;

460  stan::gm::list\_argument\* metric

461  = dynamic\_cast<stan::gm::list\_argument\*>(algo->arg("hmc")->arg("metric"));

462  if (metric->value() == "unit\_e") {

463  metric\_index = 0;

464  } else if (metric->value() == "diag\_e") {

465  metric\_index = 1;

466  } else if (metric->value() == "dense\_e") {

467  metric\_index = 2;

468  }

469

470  int sampler\_select = engine\_index

471  + 10 \* metric\_index

472  + 100 \* static\_cast<int>(adapt\_engaged);

473

474  switch (sampler\_select) {

475

476  case 0: {

477  typedef stan::mcmc::unit\_e\_static\_hmc<Model, rng\_t> sampler;

478  sampler\_ptr = new sampler(model, base\_rng, &std::cout, &std::cout);

479  if (!init\_static\_hmc<sampler>(sampler\_ptr, algo)) return 0;

480  break;

481  }

482

483  case 1: {

484  typedef stan::mcmc::unit\_e\_nuts<Model, rng\_t> sampler;

485  sampler\_ptr = new sampler(model, base\_rng, &std::cout, &std::cout);

486  if (!init\_nuts<sampler>(sampler\_ptr, algo)) return 0;

487  break;

488  }

489

490  case 10: {

491  typedef stan::mcmc::diag\_e\_static\_hmc<Model, rng\_t> sampler;

492  sampler\_ptr = new sampler(model, base\_rng, &std::cout, &std::cout);

493  if (!init\_static\_hmc<sampler>(sampler\_ptr, algo)) return 0;

494  break;

495  }

496

497  case 11: {

498  typedef stan::mcmc::diag\_e\_nuts<Model, rng\_t> sampler;

499  sampler\_ptr = new sampler(model, base\_rng, &std::cout, &std::cout);

500  if (!init\_nuts<sampler>(sampler\_ptr, algo)) return 0;

501  break;

502  }

503

504  case 20: {

505  typedef stan::mcmc::dense\_e\_static\_hmc<Model, rng\_t> sampler;

506  sampler\_ptr = new sampler(model, base\_rng, &std::cout, &std::cout);

507  if (!init\_static\_hmc<sampler>(sampler\_ptr, algo)) return 0;

508  break;

509  }

510

511  case 21: {

512  typedef stan::mcmc::dense\_e\_nuts<Model, rng\_t> sampler;

513  sampler\_ptr = new sampler(model, base\_rng, &std::cout, &std::cout);

514  if (!init\_nuts<sampler>(sampler\_ptr, algo)) return 0;

515  break;

516  }

517

518  case 100: {

519  typedef stan::mcmc::adapt\_unit\_e\_static\_hmc<Model, rng\_t> sampler;

520  sampler\_ptr = new sampler(model, base\_rng, &std::cout, &std::cout);

521  if (!init\_static\_hmc<sampler>(sampler\_ptr, algo)) return 0;

522  if (!init\_adapt<sampler>(sampler\_ptr, adapt, cont\_params)) return 0;

523  break;

524  }

525

526  case 101: {

527  typedef stan::mcmc::adapt\_unit\_e\_nuts<Model, rng\_t> sampler;

528  sampler\_ptr = new sampler(model, base\_rng, &std::cout, &std::cout);

529  if (!init\_nuts<sampler>(sampler\_ptr, algo)) return 0;

530  if (!init\_adapt<sampler>(sampler\_ptr, adapt, cont\_params)) return 0;

531  break;

532  }

533

534  case 110: {

535  typedef stan::mcmc::adapt\_diag\_e\_static\_hmc<Model, rng\_t> sampler;

536  sampler\_ptr = new sampler(model, base\_rng, &std::cout, &std::cout);

537  if (!init\_static\_hmc<sampler>(sampler\_ptr, algo)) return 0;

538  if (!init\_windowed\_adapt<sampler>(sampler\_ptr, adapt, num\_warmup, cont\_params))

539  return 0;

540  break;

541  }

542

543  case 111: {

544  typedef stan::mcmc::adapt\_diag\_e\_nuts<Model, rng\_t> sampler;

545  sampler\_ptr = new sampler(model, base\_rng, &std::cout, &std::cout);

546  if (!init\_nuts<sampler>(sampler\_ptr, algo)) return 0;

547  if (!init\_windowed\_adapt<sampler>(sampler\_ptr, adapt, num\_warmup, cont\_params))

548  return 0;

549  break;

550  }

551

552  case 120: {

553  typedef stan::mcmc::adapt\_dense\_e\_static\_hmc<Model, rng\_t> sampler;

554  sampler\_ptr = new sampler(model, base\_rng, &std::cout, &std::cout);

555  if (!init\_static\_hmc<sampler>(sampler\_ptr, algo)) return 0;

556  if (!init\_windowed\_adapt<sampler>(sampler\_ptr, adapt, num\_warmup, cont\_params))

557  return 0;

558  break;

559  }

560

561  case 121: {

562  typedef stan::mcmc::adapt\_dense\_e\_nuts<Model, rng\_t> sampler;

563  sampler\_ptr = new sampler(model, base\_rng, &std::cout, &std::cout);

564  if (!init\_nuts<sampler>(sampler\_ptr, algo)) return 0;

565  if (!init\_windowed\_adapt<sampler>(sampler\_ptr, adapt, num\_warmup, cont\_params))

566  return 0;

567  break;

568  }

569

570  default:

571  std::cout << "No sampler matching HMC specification!" << std::endl;

572  return 0;

573  }

574

575  }

576

577  // Headers

578  writer.write\_sample\_names(s, sampler\_ptr, model);

579  writer.write\_diagnostic\_names(s, sampler\_ptr, model);

580

581  std::string prefix = "";

582  std::string suffix = "\n";

583  NoOpFunctor startTransitionCallback;

584

585  // Warm-Up

586  clock\_t start = clock();

587

588  warmup<Model, rng\_t>(sampler\_ptr, num\_warmup, num\_samples, num\_thin,

589  refresh, save\_warmup,

590  writer,

591  s, model, base\_rng,

592  prefix, suffix, std::cout,

593  startTransitionCallback);

594

595  clock\_t end = clock();

596  warmDeltaT = (double)(end - start) / CLOCKS\_PER\_SEC;

597

598  if (adapt\_engaged) {

599  dynamic\_cast<mcmc::base\_adapter\*>(sampler\_ptr)->disengage\_adaptation();

600  writer.write\_adapt\_finish(sampler\_ptr);

601  }

602

603  // Sampling

604  start = clock();

605

606  stan::common::sample<Model, rng\_t>(sampler\_ptr, num\_warmup, num\_samples, num\_thin,

607  refresh, true,

608  writer,

609  s, model, base\_rng,

610  prefix, suffix, std::cout,

611  startTransitionCallback);

612

613  end = clock();

614  sampleDeltaT = (double)(end - start) / CLOCKS\_PER\_SEC;

615

616  writer.write\_timing(warmDeltaT, sampleDeltaT);

617

618  if (sampler\_ptr) delete sampler\_ptr;

619

620  }

621

622  if (output\_stream) {

623  output\_stream->close();

624  delete output\_stream;

625  }

626

627  if (diagnostic\_stream) {

628  diagnostic\_stream->close();

629  delete diagnostic\_stream;

630  }

631

632  for (size\_t i = 0; i < valid\_arguments.size(); ++i)

633  delete valid\_arguments.at(i);

634

635  return 0;

636

637  }

638

639  } // namespace common

640

641 } // namespace stan

642

643 #endif

stan::gm::singleton\_argument::set\_value

bool set\_value(const T &value)

**Definition:** singleton\_argument.hpp:126

stan::gm::argument\_parser::parse\_args

int parse\_args(int argc, const char \*argv[], std::ostream \*out=0, std::ostream \*err=0)

**Definition:** argument\_parser.hpp:28

stan::common::dump\_factory

**Definition:** context\_factory.hpp:19

stan::agrad::gradient

void gradient(const F &f, const Eigen::Matrix< double, Eigen::Dynamic, 1 > &x, double &fx, Eigen::Matrix< double, Eigen::Dynamic, 1 > &grad\_fx)

Calculate the value and the gradient of the specified function at the specified argument.

**Definition:** autodiff.hpp:93

stan::mcmc::adapt\_diag\_e\_nuts

**Definition:** adapt\_diag\_e\_nuts.hpp:16

stan::agrad::fabs

fvar< T > fabs(const fvar< T > &x)

**Definition:** fabs.hpp:18

stan::common::NoOpFunctor::operator()

void operator()()

**Definition:** command.hpp:63

stan::gm::argument\_parser::help\_printed

bool help\_printed()

**Definition:** argument\_parser.hpp:215

csv.hpp

write\_iteration\_csv.hpp

arg\_random.hpp

stan::gm::arg\_random

**Definition:** arg\_random.hpp:12

warmup.hpp

stan::gm::error\_codes::SOFTWARE

**Definition:** error\_codes.hpp:15

run\_markov\_chain.hpp

stan::common::write\_stan

void write\_stan(std::ostream \*s, const std::string prefix="")

**Definition:** write\_stan.hpp:12

stan::gm::argument\_parser

**Definition:** argument\_parser.hpp:17

write\_stan.hpp

stan::gm::list\_argument::arg

argument \* arg(std::string name)

**Definition:** list\_argument.hpp:151

adapt\_unit\_e\_nuts.hpp

stan::gm::argument::description

std::string description() const

**Definition:** argument.hpp:30

stan::mcmc::base\_adapter

**Definition:** base\_adapter.hpp:8

stan::optimization::newton\_step

double newton\_step(M &model, std::vector< double > &params\_r, std::vector< int > &params\_i, std::ostream \*output\_stream=0)

**Definition:** newton.hpp:34

init\_nuts.hpp

adapt\_unit\_e\_static\_hmc.hpp

write\_model.hpp

print\_progress.hpp

json.hpp

stan::gm::singleton\_argument

**Definition:** singleton\_argument.hpp:44

adapt\_dense\_e\_static\_hmc.hpp

write\_error\_msg.hpp

stan::gm::categorical\_argument::arg

argument \* arg(const std::string name)

**Definition:** categorical\_argument.hpp:143

stan::gm::error\_codes::OK

**Definition:** error\_codes.hpp:11

stan::common::recorder::csv

Writes out a vector as string.

**Definition:** csv.hpp:19

stan::model::gradient

void gradient(const M &model, const Eigen::Matrix< double, Eigen::Dynamic, 1 > &x, double &f, Eigen::Matrix< double, Eigen::Dynamic, 1 > &grad\_f, std::ostream \*msgs=0)

**Definition:** util.hpp:405

do\_bfgs\_optimize.hpp

adapt\_dense\_e\_nuts.hpp

stan::gm::categorical\_argument

**Definition:** categorical\_argument.hpp:11

argument\_parser.hpp

stan::gm::singleton\_argument::value

T value()

**Definition:** singleton\_argument.hpp:124

stan::gm::argument\_parser::arg

argument \* arg(std::string name)

**Definition:** argument\_parser.hpp:207

stan::mcmc::unit\_e\_nuts

**Definition:** unit\_e\_nuts.hpp:17

stan::gm::list\_argument

**Definition:** list\_argument.hpp:12

arg\_data.hpp

stan::mcmc::adapt\_unit\_e\_static\_hmc

**Definition:** adapt\_unit\_e\_static\_hmc.hpp:17

stan::common::write\_error\_msg

void write\_error\_msg(std::ostream \*error\_stream, const std::exception &e)

**Definition:** write\_error\_msg.hpp:11

bfgs.hpp

arg\_id.hpp

cmd\_line.hpp

stan::mcmc::adapt\_dense\_e\_static\_hmc

**Definition:** adapt\_dense\_e\_static\_hmc.hpp:17

util.hpp

stan::mcmc::diag\_e\_nuts

**Definition:** diag\_e\_nuts.hpp:17

stan::gm::arg\_init

**Definition:** arg\_init.hpp:10

sample.hpp

initialize\_state.hpp

stan::common::write\_iteration

void write\_iteration(std::ostream &output\_stream, Model &model, RNG &base\_rng, double lp, std::vector< double > &cont\_vector, std::vector< int > &disc\_vector)

**Definition:** write\_iteration.hpp:19

context\_factory.hpp

write\_iteration.hpp

stan::mcmc::fixed\_param\_sampler

**Definition:** fixed\_param\_sampler.hpp:14

stan::gm::error\_codes::CONFIG

**Definition:** error\_codes.hpp:16

stan::gm::arg\_output

**Definition:** arg\_output.hpp:14

stan::common::recorder::messages

**Definition:** messages.hpp:11

newton.hpp

stan::common::var\_context\_factory

**Definition:** context\_factory.hpp:11

stan::common::do\_bfgs\_optimize

int do\_bfgs\_optimize(ModelT &model, BFGSOptimizerT &bfgs, RNGT &base\_rng, double &lp, std::vector< double > &cont\_vector, std::vector< int > &disc\_vector, std::fstream \*output\_stream, std::ostream \*notice\_stream, bool save\_iterations, int refresh, StartIterationCallback &callback)

**Definition:** do\_bfgs\_optimize.hpp:21

dump.hpp

stan::gm::arg\_id

**Definition:** arg\_id.hpp:10

stan::common::write\_model

void write\_model(std::ostream \*s, const std::string model\_name, const std::string prefix="")

**Definition:** write\_model.hpp:11

stan::mcmc::unit\_e\_static\_hmc

**Definition:** unit\_e\_static\_hmc.hpp:18

mcmc\_writer.hpp

stan::gm::singleton\_argument::is\_default

bool is\_default()

**Definition:** singleton\_argument.hpp:144

stan::math::e

double e()

Return the base of the natural logarithm.

**Definition:** constants.hpp:86

stan::mcmc::adapt\_diag\_e\_static\_hmc

**Definition:** adapt\_diag\_e\_static\_hmc.hpp:17

adapt\_diag\_e\_static\_hmc.hpp

stan::gm::arg\_data

**Definition:** arg\_data.hpp:12

arg\_output.hpp

stan::io::mcmc\_writer

mcmc\_writer writes out headers and samples

**Definition:** mcmc\_writer.hpp:29

arg\_init.hpp

do\_print.hpp

stan::mcmc::dense\_e\_nuts

**Definition:** dense\_e\_nuts.hpp:17

init\_windowed\_adapt.hpp

adapt\_diag\_e\_nuts.hpp

stan::optimization::BFGSLineSearch

**Definition:** bfgs.hpp:394

stan::mcmc::dense\_e\_static\_hmc

**Definition:** dense\_e\_static\_hmc.hpp:18

stan::common::command

int command(int argc, const char \*argv[])

**Definition:** command.hpp:67

fixed\_param\_sampler.hpp

stan::mcmc::adapt\_dense\_e\_nuts

**Definition:** adapt\_dense\_e\_nuts.hpp:16

stan::gm::argument::arg

virtual argument \* arg(const std::string name)

**Definition:** argument.hpp:67

stan::common::NoOpFunctor

**Definition:** command.hpp:62

stan::io::dump

Represents named arrays with dimensions.

**Definition:** dump.hpp:902

stan::gm::list\_argument::value

std::string value()

**Definition:** list\_argument.hpp:160

stan::mcmc::adapt\_unit\_e\_nuts

**Definition:** adapt\_unit\_e\_nuts.hpp:16

stan::mcmc::sample

**Definition:** sample.hpp:13

init\_adapt.hpp

stan::mcmc::diag\_e\_static\_hmc

**Definition:** diag\_e\_static\_hmc.hpp:18

version.hpp

stan::gm::argument\_parser::print

void print(std::ostream \*s, const std::string prefix="")

**Definition:** argument\_parser.hpp:138

init\_static\_hmc.hpp

messages.hpp

stan::mcmc::base\_mcmc

**Definition:** base\_mcmc.hpp:13


---

[ Stan Home Page ]

*© 2011–2014,
Stan Development Team.*
